# Fibroblast Growth Factor 2-Immobilized Biointerfaces Drive Exogenous Transforming Growth Factor β1-Independent Chondrogenesis of Human Mesenchymal Stem Cell and Ectopic Cartilage Tissue Formation

**DOI:** 10.34133/bmr.0367

**Published:** 2026-06-01

**Authors:** Ji Hoon Jeong, Jae Hong Park, Myung Jin Ban, Yeongrok Lee, Hyeongtae Kim, Yura Kim, Gahyun Kim, Sai-Won Kwon, Ju Hun Lee, Sang-Heon Kim, Yongsung Hwang

**Affiliations:** ^1^ Soonchunhyang Institute of Medi-Bio Science (SIMS), Soonchunhyang University, Cheonan 31151, Chungnam-do, Republic of Korea.; ^2^ Department of Integrated Biomedical Science, Soonchunhyang University, Asan 31538, Chungnam-do, Republic of Korea.; ^3^ Department of Otorhinolaryngology–Head and Neck Surgery, College of Medicine, Soonchunhyang University Cheonan Hospital, Cheonan 31151, Republic of Korea.; ^4^ Department of Orthopaedic Surgery, College of Medicine, Soonchunhyang University Cheonan Hospital, Cheonan 31151, Republic of Korea.; ^5^ Department of Bionano Engineering, Center for Bionano Intelligence Education and Research, Hanyang University, Ansan 15588, Republic of Korea.; ^6^ Center for Biomaterials, Biomedical Research Institute, Korea Institute of Science and Technology, Seoul 02792, Republic of Korea.; ^7^ Department of Bio-Med Engineering, KIST School, Korea University of Science and Technology, Seoul 02792, Republic of Korea.

## Abstract

Articular cartilage regeneration remains challenging due to limited intrinsic repair capacity and the tendency of current human mesenchymal stem cell (hMSC)-based strategies to form fibrocartilage or undergo hypertrophy. Here, we present a defined biomaterial platform in which maltose-binding protein–fibroblast growth factor 2 (MBP-FGF2) is surface-immobilized to create a bioinstructive interface that directs in vitro hMSC chondrogenesis without exogenous transforming growth factor-β1 (TGF-β1). The immobilized FGF2 interface promotes spontaneous, scaffold-free spheroid assembly via FGFR1–heparan sulfate proteoglycan-mediated adhesion, providing localized and sustained signaling. Transcriptomic and molecular analyses revealed early activation of FGF- and TGF-β-related pathways, with up-regulation of SOX9, COL2A1, and aggrecan and suppression of COL1A1 and COL10A1. These effects were consistently observed in hMSCs derived from both human embryonic stem cells and human tonsil tissue. Upon subcutaneous implantation into immunodeficient mice, pre-differentiated spheroids generated ectopic cartilage tissue rich in type II collagen and aggrecan, even in the absence of TGF-β1. Taken together, these findings establish FGF2-immobilized biointerfaces as bioinstructive matrices that enable exogenous TGF-β-independent chondrogenesis and ectopic cartilage tissue formation, providing a defined and reproducible route for generating chondrogenic constructs for regenerative medicine.

## Introduction

Degeneration of articular cartilage has profound clinical consequences, largely due to the poor self-repair capacity and lack of effective regenerative treatments [1]. Current clinical interventions, such as microfracture or autologous chondrocyte implantation, often lead to the formation of fibrocartilage, which lacks the biomechanical integrity of native hyaline cartilage and is prone to further degradation [2]. Therefore, cartilage tissue engineering has emerged as a promising strategy to restore functional cartilage. Mesenchymal stem cells (MSCs) are widely studied for cartilage regeneration, due to their multipotency and robust proliferative capacity. They can differentiate into various mesenchymal lineages, such as cartilage, bone, and adipose tissue [3]. Accordingly, several in vitro culture methods have been developed to promote the chondrogenic differentiation of MSCs, such as monolayer expansion, pellet culture via centrifugation [4,5], high-density micromass culture [6], and biomaterial-based scaffold systems [7].

Pellet culture using defined chondrogenic media supplemented with transforming growth factor-β (TGF-β) is a standard in vitro model for inducing chondrogenesis in MSCs [4,8]. This method provides a 3-dimensional (3D) environment that mimics pre-cartilage condensation during embryogenesis and facilitates cell–cell interactions that induce the expression of cartilage markers, such as type II collagen and aggrecan. Nevertheless, achieving consistent, stable chondrogenic differentiation remains a major challenge. Extended exposure to TGF-β isoforms, although effective at inducing lineage commitment, frequently results in unwanted fibrocartilage features, such as type 1 collagen expression and hypertrophic maturation, characterized by an up-regulation of type X collagen, ultimately producing unstable or ossifying cartilage [9]. These differentiated MSC pellets commonly undergo hypertrophy and terminal differentiation following ectopic transplantation [10], in contrast with the stable phenotype of native hyaline chondrocytes.

Fibroblast growth factor 2 (FGF2), a member of the heparin-binding FGF family, is a potent mitogen for various mesenchymal cell types [11]. It has emerged as a key regulator of MSC proliferation and chondrogenic lineage priming [12]. Beyond its mitogenic effects, FGF2 is instrumental in regulating signaling pathways essential for cartilage development and maintenance [13]. In vitro studies have shown that short-term FGF2 preconditioning significantly elevates the levels of basal SOX9 protein, a crucial transcription factor in chondrogenesis, thereby enhancing the chondrogenic differentiation capacity of MSCs [12]. When applied during monolayer expansion, FGF2 improves subsequent 3D chondrogenesis, as evidenced by increased glycosaminoglycan (GAG) production and elevated expression of type II collagen and aggrecan [14]. Additionally, FGF2 has been shown to enhance the redifferentiation of dedifferentiated chondrocytes and restore the expression of key cartilage markers, including type II collagen, in pellet cultures. These effects underscore its dual capacity to preserve progenitor traits and modulate lineage-specific transcriptional programs [15]. Collectively, these findings position FGF2 as a compelling driver of stable and efficient chondrogenesis with considerable promise for cartilage tissue engineering.

Recent advances in biomaterials have aimed to replicate features of the native stem cell niche by integrating well-defined physical and biochemical cues [16]. Among these, surface-immobilized growth factors have gained attention as a strategy for achieving localized, receptor-specific signaling while minimizing off-target effects associated with soluble cytokines [17,18]. A notable development is the maltose-binding protein-tagged FGF2 (MBP-FGF2), a fusion protein that stably adsorbs to polystyrene (PS) substrates [19]. Unlike soluble FGF2, immobilized MBP-FGF2 enables sustained and spatially confined engagement with FGF receptors and heparan sulfate proteoglycans (FGFR–HSPG) while reducing integrin-mediated adhesion. This promotes the spontaneous self-assembly of MSCs into compact spheroids without the need for external forces such as centrifugation [20]. The self-assembly process is driven by receptor-specific FGF2–FGFR interactions, stabilized by HSPGs, alongside reduced integrin adhesion and enhanced cell–cell interactions, mimicking key aspects of developmental mesenchymal condensation [21]. This biomaterial-induced spheroid formation recapitulates condensation-like architecture and activates endogenous TGF-β3 signaling, priming cells for chondrogenic differentiation in the absence of exogenous TGF-β1. Spheroids generated on MBP-FGF2-coated surfaces demonstrate robust deposition of cartilage-specific matrix components while suppressing hypertrophic markers, highlighting the translational potential of this defined, scaffold-free platform for engineering stable, hyaline-like cartilage.

## Materials and Methods

### Preparation and culture of H9-derived MSCs

Human H9 embryonic stem cell (H9)-derived MSCs (H9-MSCs) were used in this study. The H9 human embryonic stem cell line was obtained from the WiCell Research Institute (Madison, WI, USA) and used in accordance with the provider’s guidelines and applicable ethical regulations. The culture and differentiation protocols were as follows: H9 embryonic stem cells were initially cultured using a feeder-based system. Mouse embryonic fibroblasts (MEFs) were seeded onto 0.1% gelatin-coated cell culture plates and incubated for 1 d in Dulbecco’s modified Eagle’s medium [DMEM; 10-013-CVR, high glucose (HG), Corning, NY, USA] supplemented with 10% fetal bovine serum (FBS; 35-010-CV, Corning) and 1% penicillin–streptomycin (P/S; 15140163, Gibco, Waltham, MA, USA). H9 cells were seeded on MEFs until confluence and cultured in KnockOut DMEM (10829-018, Gibco) supplemented with 20% KnockOut Serum Replacement (10828-028, Gibco), 1× P/S, 1% GlutaMAX (35050061, Gibco), 1% nonessential amino acids (11140-050, Gibco), and 0.5% β-mercaptoethanol (21985-023, Gibco). The culture medium was refreshed daily, and cells were incubated at 37 °C in a 5% CO₂ humidified atmosphere. Cells were detached using accutase (A11105-01, Gibco) and seeded on 0.1% gelatin-coated plates for 15 min to remove MEFs (Corning). The resulting cell population was then seeded on ultra-low attachment plates to form embryoid bodies (EBs) for 1 week. The formed EBs were then transferred to Matrigel-coated plates (354234, Corning) and cultured for 1 week in high-glucose DMEM (DMEM-HG) supplemented with 10% FBS and 1% P/S. After this differentiation phase, cells were detached using accutase, filtered using a 70-μm cell strainer (93070, SPL Life Science, Pocheon, Korea), and seeded onto standard cell culture plates. They were maintained in DMEM-HG supplemented with 10% FBS and 1× P/S at 37 °C in a 5% CO₂ humidified atmosphere.

### Isolation and culture of tonsil-derived MSCs

Tonsil-derived MSCs (T-MSCs) were isolated from human tonsil tissue samples obtained from patients undergoing surgery. The study protocol was reviewed and approved by the Institutional Review Board of Soonchunhyang University Hospital, Cheonan, Korea (protocol no. SCHCA IRB 2018-01-019). Informed consent was obtained from all participants prior to the surgery. Harvested tonsil tissue was washed with phosphate-buffered saline (PBS), minced into pieces of approximately 1 mm^3^, and enzymatically digested in DMEM-HG containing collagenase type I (17100-017, Gibco) for 1 h at 37 °C. After enzymatic digestion, the suspension was diluted with DMEM-HG supplemented with 10% FBS and 1% P/S and then filtered using a 100-μm cell strainer. The filtrate was centrifuged at 300*g* for 5 min, and the resulting cell pellet was washed twice with PBS. Cells were further purified via density gradient centrifugation using Ficoll (density: 1.073), followed by additional PBS washes. Then, the isolated T-MSCs were seeded and cultured in DMEM-HG supplemented with 10% FBS and 1% P/S at 37 °C in a humidified incubator at 5% CO_2_.

### Flow cytometry analysis

H9-MSCs and T-MSCs were incubated with the following fluorochrome-conjugated antibodies (all from BioLegend, San Diego, CA, USA): phycoerythrin (PE) anti-human CD166 (343904), fluorescein isothiocyanate (FITC) anti-human CD73 (344016), FITC anti-human CD105 (323204), PE anti-human CD29 (303004), FITC anti-mouse/human CD44 (103022), PE anti-human CD31 (303106), and Alexa Fluor 488 anti-human CD34 (343518). All antibodies were diluted in PBS to a ratio of 1:20 prior to staining.

### Preparation of MBP-FGF2 and MBP-FGF2-immobilized surfaces

To generate an FGF2-immobilized surface, 48-well nontreated PS plates were coated with MBP-FGF2, as previously described [21]. Briefly, the MBP-FGF2 fusion protein was expressed in *Escherichia coli* harboring a pMAL-bFGF plasmid, which was generated by inserting human FGF2 complementary DNA (cDNA; Bioneer, Daejeon, Korea) into a pMAL expression vector (New England Biolabs, Ipswich, MA, USA). For MBP-FGF2 immobilization on the surface, MBP-FGF2 was diluted in the PBS to a final concentration of 10 μg/ml. A total volume of 200 μl of the MBP-FGF2 solution was added to each well and incubated at room temperature for 4 h under sterile conditions to allow adsorption. After incubation, wells were rinsed 3 times with sterile PBS to remove unbound MBP-FGF2. To minimized nonspecific cell adhesion, the coated surfaces were blocked with 1% bovine serum albumin (BSA; SM-BOV-100, Geneall, Seoul, Korea) in PBS for 1 h at room temperature, followed by gentle washing with PBS prior to cell seeding. MBP-FGF2 coating was performed freshly immediately before cell seeding.

### Cell culture and chondrogenic differentiation on MBP-FGF2-immobilized surfaces

Cells were seeded onto MBP-FGF2-coated surfaces at a density of 1 × 10^5^ cells and cultured in DMEM-HG supplemented with 10% FBS and 1% P/S for 48 h, during which they spontaneously assembled into uniform spheroids. After spheroid formation, the culture medium was replaced with chondrogenic differentiation medium, and culture was maintained for 3 weeks with medium changes every 3 d. The chondrogenic differentiation medium consisted of low-glucose DMEM (10-014-CV, Corning) supplemented with 100 nM dexamethasone (D2915, Sigma, St. Louis, MO, USA), 40 μg/ml proline (PRO222, Bioshop, Hanam, Korea), 50 μg/ml ascorbic acid-2-phosphate (A8960, Sigma), 100 μg/ml sodium pyruvate (11360-070, Gibco), 1× ITS (insulin–transferrin–selenium) + Premix (354352, Corning), and 1% P/S, with or without 10 ng/ml TGF-β1 (30R-AT027, Fitzgerald, Acton, MA, USA).

### Total RNA extraction

Total RNA was extracted using TRIzol reagent (15596018, Invitrogen, Carlsbad, CA, USA) according to the manufacturer’s protocol. Chondrogenic spheroids were harvested after 1 and 3 weeks of differentiation and homogenized in TRIzol. Following complete cell lysis, chloroform was added, and the mixture was mixed vigorously and incubated at room temperature for 5 min. Samples were centrifuged at 14,000*g* for 20 min, and the aqueous phase was carefully collected. Isopropanol (500 μl) was added to the supernatant, mixed thoroughly, and incubated for 5 min at room temperature. The mixture was then centrifuged again at 14,000*g* for 20 min. After discarding the supernatant, the RNA pellet was washed with 1 ml of 75% ethanol [prepared with diethyl pyrocarbonate (DEPC)-treated water], vortexed briefly, and centrifuged at 10,000*g* for 10 min. The pellet was air-dried for 10 min and resuspended in DEPC-treated water. The RNA solution was incubated at 55 °C for 10 min on a heating block to ensure complete dissolution. RNA concentration and purity were assessed using a NanoDrop spectrophotometer.

### RNA sequencing and data analysis

RNA samples were collected at 0 and 4 h after seeding on MBP-FGF2-immobilized surfaces and sent to Beijing Genomics Institute (China) for library preparation and sequencing. RNA-sequencing (RNA-seq) libraries were constructed using a SMARTer mRNA-Seq Library Prep Kit, according to the manufacturer’s instructions. Sequencing was performed using the HiSeq 2500 platform (Illumina, San Diego, CA, USA), generating >20 million strand-specific paired-end reads (101 base pairs) per sample. Clean reads were aligned to the human reference genome using Hierarchical Indexing for Spliced Alignment of Transcripts 2 (HISAT2). Differentially expressed genes were identified based on fragments per kilobase of transcript per million mapped reads values, with a fold change > 1.3 and *P* < 0.05 for comparisons at 4 h relative to 0 h. All expression data were normalized to the corresponding 0-h samples prior to downstream analyses. Gene Ontology (GO) analysis, including biological process, cellular components, molecular functions, and Kyoto Encyclopedia of Genes and Genomes (KEGG) pathway enrichment analysis were performed using the DAVID bioinformatics resource (https://davidbioinformatics.nih.gov/). Hierarchical clustering and heat map analyses were performed using the MeV software package. Gene Set Enrichment Analysis (GSEA) was performed using GSEA software (v4.1.0, Broad Institute, Cambridge, MA, USA) using default parameters. Ranked gene lists based on expression fold changes at 4 h relative to 0 h were used as input. The Molecular Signatures Database (MSigDB v7.4) was employed as the reference set, and enrichment scores with a nominal *P* < 0.05 and false discovery rate < 0.25 were considered significant. Volcano plots were generated using R software (v4.2.0) in RStudio (Posit, Boston, MA, USA). Differential expression results were visualized using the Enhanced Volcano package, with thresholds for significance set at fold change > 2 and an adjusted *P* < 0.05.

### Quantitative real-time PCR analysis

Total RNA was extracted using TRIzol reagent and reverse-transcribed into cDNA using the ReverTra Ace qPCR RT master mix with gDNA Remover (FSQ-301, Toyobo, Osaka, Japan), following the manufacturer’s protocol. Quantitative real-time polymerase chain reaction (qRT-PCR) was performed using the SYBR Green Real-time PCR master mix (4472918, Toyobo) on a QuantStudio Real-Time PCR system (Applied Biosystems, Foster City, CA, USA). Gene expression levels were calculated using the 2^−ΔΔCt^ method, with glyceraldehyde-3-phosphate dehydrogenase as the reference gene. Primer sequences used for amplification of target genes are listed in Table S1.

### Immunohistochemistry and histological staining

Chondrogenic spheroids were fixed in 4% paraformaldehyde (CNP015-0500, Cell Nest, Tokyo, Japan) for 10 min at room temperature and washed 3 times with PBS. For cryoprotection, spheroids were incubated in 20% (w/v) sucrose (Suc507.1, Bioshop) in 1× PBS overnight at 4 °C, followed by incubation with optimal cutting temperature (OCT) compound (KMA-0100-00A, Cell Path, Powys, UK) at a 1:1 ratio. Samples were then immersed in 100% OCT for 4 h, embedded in cryomolds, and rapidly frozen in isopentane cooled with liquid nitrogen. Embedded samples were stored at −80 °C until sectioning. Frozen spheroids were sectioned into 15-μm-thick sections using a cryostat (Leica, Wetzlar, Germany) and mounted on adhesive glass slides (APS-11, MATSUNAMI, Osaka, Japan). For Alcian blue staining, sections were first washed with deionized (DI) water for 5 min to remove residual OCT. Sections were then stained with freshly prepared Alcian blue solution, consisting of 0.25 g of Alcian blue powder (A3157, Sigma) dissolved in 25 ml of 0.1 M HCl for 1 h at room temperature. After staining, slides were rinsed with DI water and sequentially dehydrated with 95% and 100% ethanol, followed by xylene treatment. Immunofluorescence staining was performed on spheroids generated from the same differentiation batch under identical culture conditions. Spheroids were fixed, sectioned, and stained at the central region. Due to limited tissue per section, multiple markers were not analyzed on the same spheroid; instead, separate spheroids from the same batch were used. All samples were processed in parallel using identical fixation, staining, and imaging conditions to minimize technical variability.

### Immunofluorescence staining

Cells and tissue sections were fixed with 4% paraformaldehyde for 10 min at room temperature. Fixed samples were blocked with 3% (w/v) BSA (ALB001.100, Bioshop) in PBS and permeabilized with 0.3% (v/v) Triton X-100 (TRX777.500, Bioshop) for 1 h at room temperature. Fixed samples were then incubated with primary antibodies diluted in 1% (w/v) BSA for 1 h at room temperature. The following primary antibodies were used: Oct3/4 (sc-5279, Santa Cruz, Dallas, TX, USA), collagen type I (1:200, ab34710, Abcam, Cambridge, UK), collagen type II (1:200, II-II6B3, Developmental Studies Hybridoma Bank, Iowa, IA, USA), collagen type X (1:200, ab58632, Abcam), SOX9 (1:200, ab185230, Abcam), TGF-β3 (1:200, sc-166861, Santa Cruz), TGF-βR3 (1:200, sc-130348, Santa Cruz), human nuclear antigen (HNA; 1:200, ab191181, Abcam), and human-specific lamin A (H-LMNA; 1:200, ab108595, Abcam). Samples were washed 3 times with PBS (5 min each) and incubated with secondary antibodies in 1% (w/v) BSA for 1 h at room temperature: anti-goat Alexa 555 (A21422, Invitrogen) and anti-mouse Alexa 488 (A11001, Invitrogen). Nuclei were counterstained with Hoechst 33342 (1 μg/ml; H21492, Molecular Probes, Eugene, CA, USA) for 10 min at room temperature. Fluorescence images were acquired using a fluorescence microscope (Evos M7000, Invitrogen) and confocal microscope (LSM 710, Carl Zeiss, Oberkochen, Germany) at the Soonchunhyang Biomedical Research Core Facility of the Korea Basic Science Institute.

### Subcutaneous transplantation of chondrocyte spheroids

Chondrocyte spheroids were embedded in a photo-crosslinkable hydrogel consisting of 2% (w/v) methacrylate hyaluronic acid (914568, Sigma) and 0.5% (w/v) Irgacure 2959 as the photoinitiator (55047962, BASF, Ludwigshafen, Germany). The spheroid-laden hydrogel precursor solution was dispensed into the cap of a 1.5-ml tube and subsequently crosslinked via ultraviolet exposure (365 nm) for 3 min. The crosslinked hydrogel constructs containing chondrocyte spheroids, pre-differentiated for 1 week with or without 10 ng/ml TGF-β1, were subcutaneously implanted into the dorsal region of 10-week-old male nonobese diabetic (NOD) severe combined immunodeficient (scid) gamma mice (catalog no. 005557, Jackson Laboratory, Bar Harbor, ME, USA) under isoflurane anesthesia. Bilateral subcutaneous pockets were generated via blunt dissection following 2 small dorsal skin incisions. Hydrogel constructs were implanted into each pocket (left: without TGF-β1; right: with TGF-β1), and the incisions were closed using sutures. Animals were maintained under standard housing conditions and monitored for 6 weeks post-implantation.

### Statistical analysis

For 2-group comparisons, statistical significance was assessed using Student’s *t* test. For multiple-group comparisons, significance was assessed using one-way analysis of variance, followed by Tukey’s multiple-comparison test, using GraphPad Prism 9.0 (GraphPad, San Diego, CA, USA). Statistical significance is indicated as *P < 0.05, **P < 0.01, and ***P < 0.001.

## Results

### MBP-FGF2-immobilized surfaces induce spontaneous formation of human MSC spheroids

**Fig. 1.**
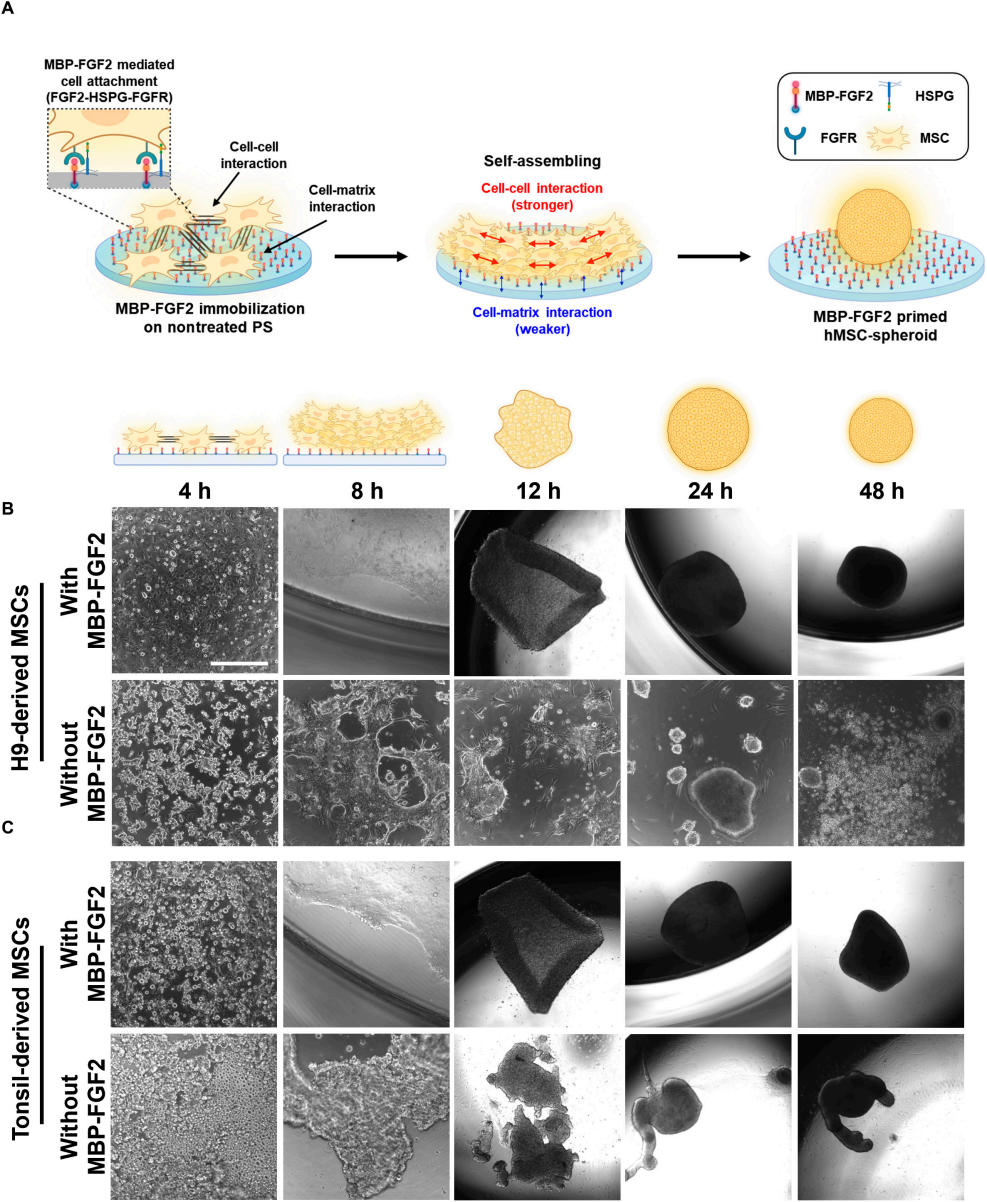
MBP-FGF2-immobilized surfaces promote self-assembly of MSC spheroids. (A) Schematic illustration of MBP-FGF2-mediated spheroid formation through FGFR–HSPG interactions, reduced cell–matrix adhesion, and enhanced cell–cell interactions. (B and C) Time-course images showing spheroid formation of H9-MSCs and T-MSCs on MBP-FGF2-immobilized surfaces versus uncoated surfaces at 4, 8, 12, 24, and 48 h (scale bar, 400 μm). MBP-FGF2, maltose-binding protein–fibroblast growth factor 2; MSC, mesenchymal stem cell; HSPG, heparan sulfate proteoglycan; FGFR, fibroblast growth factor receptor.

To establish a defined platform for scaffold-free 3D culture, we engineered PS surfaces coated with MBP-FGF2. The MBP domain enables passive adsorption onto the hydrophobic surface of nontreated PS plates, whereas the FGF2 domain promotes cellular interaction via FGFR1 on the cell surface. Moreover, immobilized MBP-FGF2 provides sustained and spatially localized signaling through stable interaction with HSPGs, thereby supporting integrin-independent cell adhesion. Under these conditions, cells adhere to the surface primarily through FGFR1-HSPG-mediated binding, resulting in relatively weak cell–substrate adhesion while promoting stronger cell–cell adhesion. This biofunctionalized surface facilitates both cell–substrate and cell–cell interactions, enabling the spontaneous self-assembly of MSCs into uniformly compact spheroids without the need for external forces, centrifugation, or low-attachment conditions (Fig. 1A). In this study, we utilized MSCs, which were derived from both H9-hESCs (human embryonic stem cells) and human tonsil tissue. Prior to differentiation of H9-hESCs into MSCs, their pluripotent status was confirmed via immunofluorescence staining for Oct3/4, a key transcription factor associated with stem cell self-renewal and pluripotency (Fig. S1A). H9-hESCs were differentiated into MSCs through EB formation and subsequent outgrowth on a Matrigel-coated plate (Fig. S1B and C). The resulting cells exhibited fibroblast-like morphology and expressed MSC surface markers, including CD105, CD73, CD29, CD44, and CD166, while they did not express hematopoietic markers CD31 and CD34 (Fig. S1D). T-MSCs were isolated through collagenase digestion and Ficoll gradient purification (Fig. S2A) and were similarly confirmed to display a typical MSC phenotype (Fig. S2B). Both H9-MSCs and T-MSCs readily attached and underwent morphological rearrangement upon seeding on MBP-FGF2-immobilized surfaces. Within 8 h, cells began to flatten and spread across the coated surface. By 12 h, the edges of the monolayer began to curl inward, forming cell sheets. These structures continued to contract and self-condense over the next 24 to 48 h into tightly packed, spherical 3D aggregates (Fig. 1B and C, upper panels). This spontaneous self-organization occurred uniformly across the culture surface and consistently produced compact spheroids. In contrast, cells seeded on control surfaces (PS plates coated with 1% BSA without MBP-FGF2) showed minimal adhesion, remaining loosely aggregated and forming irregular, fragmented structures (Fig. 1B and C, lower panels). To further distinguish the effect of immobilized MBP-FGF2 from that of soluble FGF2, additional control experiments were performed. Soluble FGF2 was tested at 5, 10, 15, and 20 ng/ml, representing commonly used concentrations for MSC culture, as well as at 2.74 μg/ml to match the FGF2 content in 10 μg/ml MBP-FGF2. In parallel, soluble MBP-FGF2 was applied at 10 μg/ml. Under all soluble conditions, cells exhibited limited substrate attachment and formed irregular, loosely aggregated clusters rather than uniform spheroids (Figs. S3 and S4). In contrast, spheroid formation occurred only on MBP-FGF2-immobilized surfaces, indicating that the immobilized presentation of FGF2, rather than soluble delivery, is required to support efficient cell adhesion and subsequent self-organization.

Collectively, these results demonstrate that MBP-FGF2 functionalization supports the spontaneous, reproducible, and scaffold-free spheroid formation of MSCs. This method provides a simple and tunable strategy to promote spheroid morphogenesis directly on conventional PS plates, establishing a robust platform for 3D stem cell culture without artificial confinement or mechanical induction.

### MBP-FGF2-guided cell adhesion primes early TGF-β signaling and chondrogenic commitment during spheroid formation

**Fig. 2.**
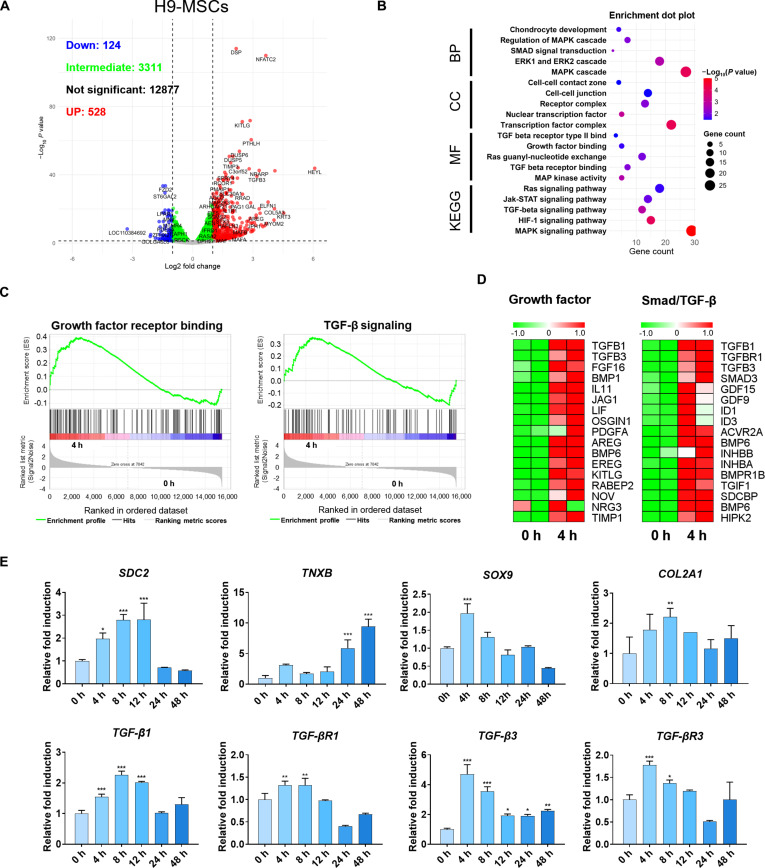
Transcriptomic and molecular validation of early chondrogenic induction in MBP-FGF2-induced H9-MSC spheroids. (A) Volcano plot showing differentially expressed genes at 4 h relative to 0 h after seeding on MBP-FGF2-imobilized surfaces in H9-MSCs. (B) GO and KEGG enrichment analyses highlighting significantly up-regulated pathways at 4 h relative to 0 h. (C) GSEA plots based on ranked gene expression changes at 4 h relative to 0 h. (D) Heatmaps showing up-regulation of growth factor- and TGF-β-related genes at 0- and 4-h samples. (E) Time-dependent qPCR analysis of genes associated with FGF2 signaling, ECM remodeling, chondrogenesis, and TGF-β signaling. Data are presented as mean ± SD. *P < 0.05; **P < 0.01; ***P < 0.001. RNA-seq analyses were performed using 2 independent biological replicates per condition (*n* = 2). All qPCR experiments were performed with 3 independent biological replicates (*n* = 3). MBP-FGF2, maltose-binding protein–fibroblast growth factor 2; MSC, mesenchymal stem cell; GO, Gene Ontology; KEGG, Kyoto Encyclopedia of Genes and Genomes; GSEA, Gene Set Enrichment Analysis; ECM, extracellular matrix; TGF-β, transforming growth factor-β; qPCR, quantitative polymerase chain reaction; SD, standard deviation.

To elucidate the molecular mechanisms involved in early spheroid formation on MBP-FGF2-immobilized surfaces, we performed transcriptomic profiling by performing RNA-seq of H9-MSCs and T-MSCs at 0 and 4 h post-seeding. For H9-MSCs, differential gene expression analysis identified 528 up-regulated and 124 down-regulated genes (fold change > 1.3, *P* < 0.05), as shown in the volcano plot (Fig. 2A). Notably, genes such as *MAP2K3* and *TGF-β3* were significantly up-regulated, indicating early activation of FGF-related and TGF-β signaling cascades. GO and KEGG pathway enrichment analyses revealed activation of biological processes related to chondrogenic differentiation, including chondrocyte development, mitogen-activated protein kinase (MAPK) cascade, and TGF-β receptor signaling (Fig. 2B). GSEA further confirmed positive enrichment of gene sets associated with growth factor receptor binding and TGF-β signaling pathway on MBP-FGF2-immobilized surfaces at 4 h (Fig. 2C). Based on the enrichment of chondrocyte development, TGF-β signaling, and growth factor-related pathways identified in GO and GSEA analyses, a heatmap was generated to visualize the expression dynamics of representative genes involved in these pathways. The results showed up-regulation of growth factors and Smad/TGF-β-related genes following spheroid formation on MBP-FGF2-immobilized surfaces (Fig. 2D). Consistent with the RNA-seq results, qRT-PCR demonstrated that the expression of key transcription factors, TGF-β pathway components, and chondrogenic markers, including TNXB and COL2A1, progressively increased over time. In addition, syndecan-2 (SDC2), an HSPG known to facilitate FGF2 signaling, was up-regulated, suggesting its involvement in early FGF2-mediated responses. Furthermore, TGF-β-related genes, including *TGF-β1*, *TGF-βR1*, *TGF-β3*, and *TGF-βR3*, and core transcription factors, such as SOX9, were dynamically up-regulated during the early phase of cell–matrix interaction, supporting the notion that MBP-FGF2-mediated cell adhesion activates TGF-β signaling and contributes to early lineage priming toward chondrogenic commitment (Fig. 2E).

**Fig. 3.**
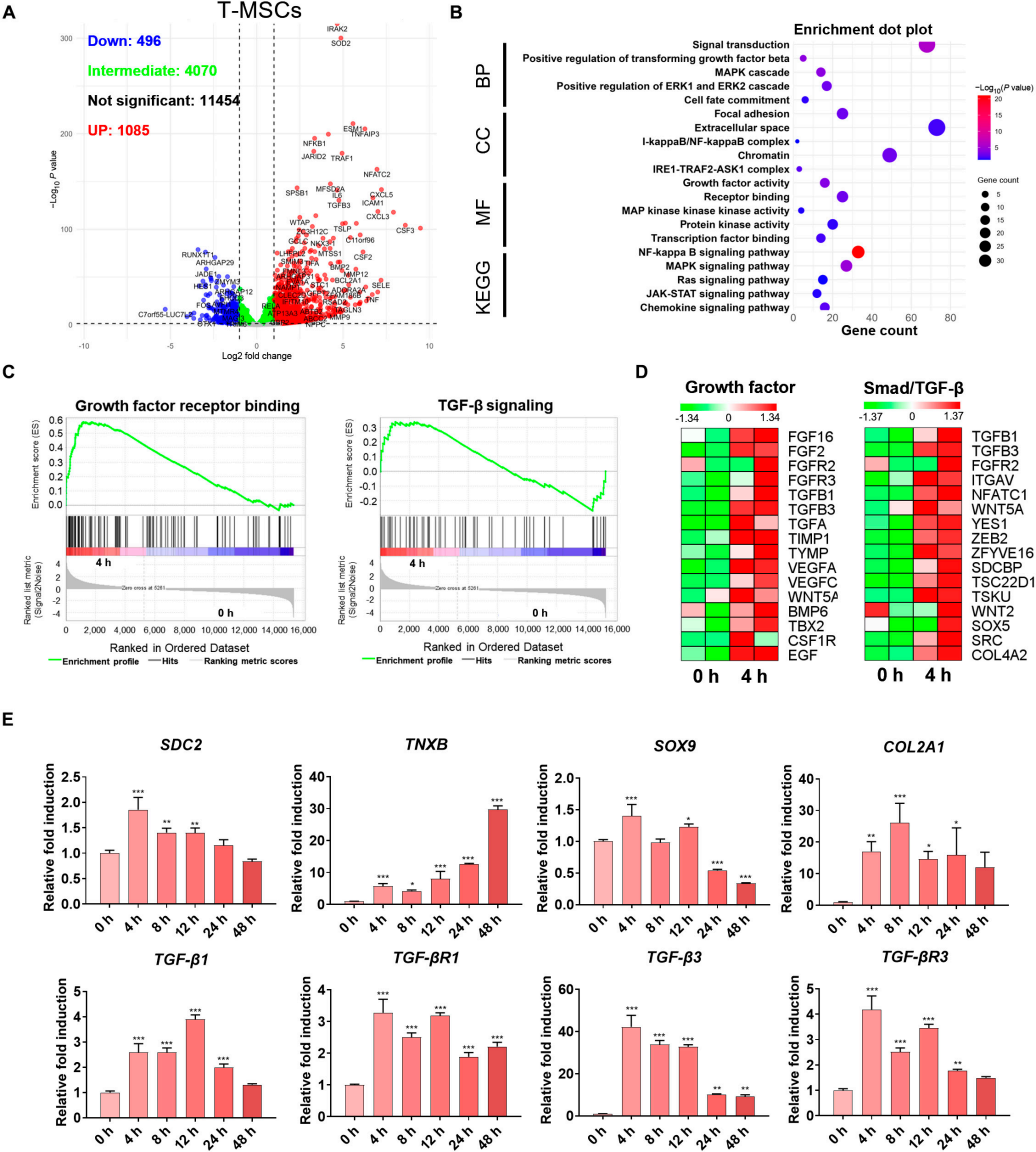
Transcriptomic and molecular validation of early chondrogenic induction in MBP-FGF2-induced T-MSC spheroids. (A) Volcano plot showing the differentially expressed genes at 4 h relative to 0 h after seeding on MBP-FGF2-imobilized surfaces in T-MSCs. (B) GO and KEGG enrichment analyses highlighting significantly up-regulated pathways at 4 h relative to 0 h. (C) GSEA plots based on ranked gene expression changes at 4 h relative to 0 h. (D) Heatmaps showing up-regulation of growth factor- and TGF-β-related genes at 0 and 4 h. (E) Time-dependent qPCR analysis of genes related to FGF2 signaling, ECM remodeling, chondrogenesis, and TGF-β signaling. Data are presented as mean ± SD. *P < 0.05; **P < 0.01; ***P < 0.001. RNA-seq analyses were performed using 2 independent biological replicates per condition (*n* = 2). All qPCR experiments were performed with 3 independent biological replicates (*n* = 3). MBP-FGF2, maltose-binding protein–fibroblast growth factor 2; MSC, mesenchymal stem cell; GO, Gene Ontology; KEGG, Kyoto Encyclopedia of Genes and Genomes; GSEA, Gene Set Enrichment Analysis; ECM, extracellular matrix; qPCR, quantitative polymerase chain reaction; TGF-β, transforming growth factor-β; SD, standard deviation.

In T-MSCs, RNA-seq revealed 1,085 up-regulated and 496 down-regulated genes at 4 h. Notably, TGF-β3, a regulator of chondrogenesis and MSC differentiation, was significantly up-regulated (Fig. 3A). Enrichment analysis identified overlapping signaling networks, including TGF-β signaling, extracellular matrix (ECM) organization, and cell fate commitment (Fig. 3B). GSEA further demonstrated strong induction of growth factor receptor binding and TGF-β signaling (Fig. 3C). Similar to H9-MSCs, T-MSCs also exhibited up-regulation of growth factors and Smad/TGF-β-related genes, indicating a conserved transcriptional response to MBP-FGF2-mediated adhesion and aggregation (Fig. 3D). qRT-PCR analysis of T-MSCs confirmed time-dependent up-regulation of key ECM and chondrogenic markers, including TNXB, SOX9, and COL2A1. In addition, SDC2, which is involved in FGF2 signaling, was up-regulated. In parallel, TGF-β1, TGF-β3, and their receptors TGF-βR1 and TGF-βR3 were up-regulated, indicating activation of TGF-β signaling and potentially driving early chondrogenic commitment during adhesion and spheroid formation on MBP-FGF2-immobilized surfaces (Fig. 3E). Together, these transcriptomic and molecular analyses demonstrate that MBP-FGF2-immobilized surfaces contribute to early lineage priming toward spheroid formation as well as trigger early activation of growth factor and TGF-β signaling cascades, thereby facilitating the initiation of chondrogenic commitment.

### MBP-FGF2-immobilized surfaces promote TGF-β1-independent chondrogenic differentiation of human MSC spheroids while suppressing hypertrophic features

Transcriptome and qRT-PCR analyses revealed that adhesion and spheroid formation on MBP-FGF2-immobilized surfaces activated TGF-β signaling and up-regulated early chondrogenic markers, including SOX9, TNXB, and COL2A1. These findings indicate that the biophysical and biochemical microenvironment established during early adhesion and spheroid assembly on MBP-FGF2-immobilized surfaces promotes chondrogenic lineage commitment.

**Fig. 4.**
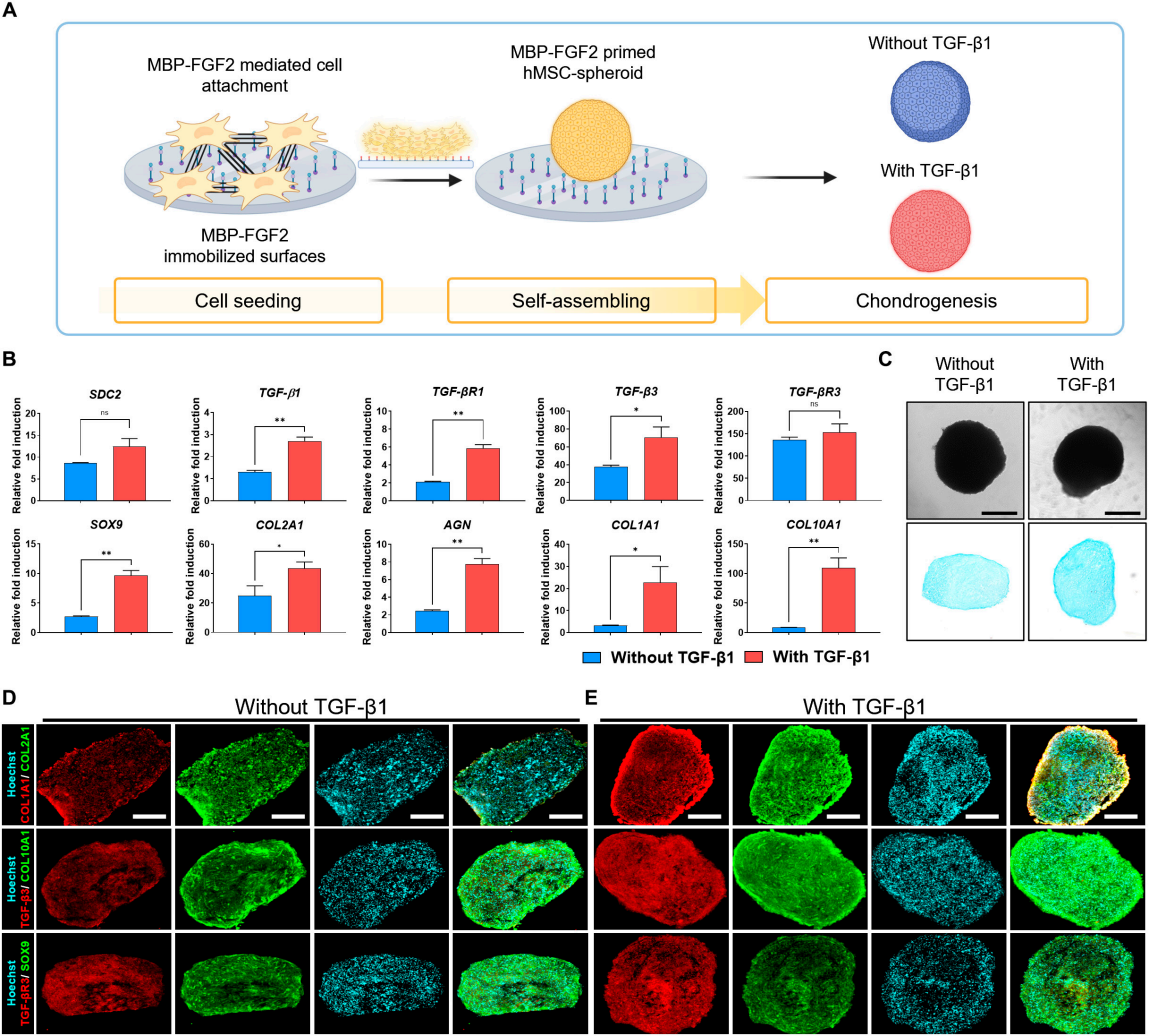
MBP-FGF2 spheroids exhibit chondrogenic differentiation with reduced hypertrophic marker expression in vitro. (A) Schematic overview of the experimental design for chondrogenic induction of MBP-FGF2-primed H9-MSC spheroids with or without TGF-β1 supplementation. (B) qPCR analysis of chondrogenic and hypertrophic marker expression in H9-MSC spheroids on day 21. (C) Alcian blue staining confirming GAG deposition in H9-MSC spheroids cultured with or without TGF-β1 (scale bar, 200 μm). (D and E) Immunofluorescence staining of H9-MSC spheroids cultured with or without TGF-β1, showing the expression of COL1A1, COL2A1, COL10A1, SOX9, TGF-β3, and TGF-βR3 (scale bar, 200 μm). Data are presented as mean ± SD. *P < 0.05; **P < 0.01; ***P < 0.001. All experiments were performed with 3 independent biological replicates (*n* = 3). MBP-FGF2, maltose-binding protein–fibroblast growth factor 2; MSC, mesenchymal stem cell; qPCR, quantitative polymerase chain reaction; TGF-β1, transforming growth factor-β1; SD, standard deviation.

To assess the chondrogenic potential of MSC spheroids formed on MBP-FGF2-immobilized surfaces, spheroids were cultured with or without exogenous TGF-β1 supplementation, and their molecular and histological profiles were analyzed overtime (Fig. 4A). Both H9-MSC and T-MSC spheroids were maintained under chondrogenic conditions for 21 d, and the expression of chondrogenic markers was evaluated through qRT-PCR and histological staining analyses. In H9-MSC chondrogenic spheroids, the expression of chondrogenic markers, including SOX9, COL2A1, AGN, TGF-β1, and TGF-β3 and their receptors, was significantly up-regulated in both groups, regardless of TGF-β1 supplementation. SDC2 expression was also higher in spheroids than in undifferentiated cells. Even without exogenous TGF-β1, a notable induction of TGF-β1, TGF-β3, and their receptors was observed. Although the expression of chondrogenic markers was lower than in the TGF-β1-treated group, hypertrophic marker expression differed substantially depending on TGF-β1 supplementation. COL1A1 and COL10A1, both associated with fibrocartilage formation and hypertrophy, were strongly up-regulated in the presence of exogenous TGF-β1. In contrast, their expression was reduced in its absence, indicating a cartilage-like phenotype with attenuated fibrocartilage and hypertrophy (Fig. 4B). Alcian blue staining confirmed GAG deposition under both conditions, with more intense staining in the TGF-β1-treated group, reflecting enhanced ECM deposition. However, GAG-positive regions were also observed in TGF-β1-untreated spheroids (Fig. 4C). To further assess matrix composition and chondrogenic differentiation at the protein level, immunofluorescence staining was performed. Staining results revealed strong COL2A1 expression colocalized with SOX9, TGF-β3, and TGF-βR3 in both groups. In contrast, COL1A1 and COL10A1 signals were weaker in spheroids cultured without TGF-β1, supporting a stable hyaline-like cartilage phenotype (Fig. 4D and E). These results demonstrate that MBP-FGF2-immoblized surface enables chondrogenic maturation while effectively suppressing hypertrophic and fibrocartilaginous differentiation in the absence of exogenous TGF-β1.

**Fig. 5.**
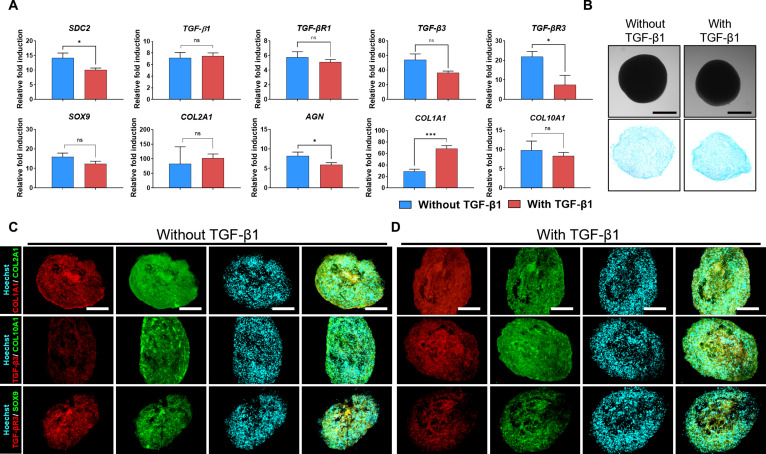
MBP-FGF2 spheroids exhibit chondrogenic differentiation with reduced hypertrophic marker expression in vitro. (A) qPCR analysis of chondrogenic and hypertrophic marker expression in T-MSC spheroids on day 21 under chondrogenic differentiation with or without TGF-β1 supplementation. (B) Alcian blue staining confirming GAG deposition in T-MSC spheroids cultured with or without TGF-β1 (scale bar, 200 μm). (C and D) Immunofluorescence staining of T-MSC spheroid cultures with or without TGF-β1, showing the expression of COL1A1, COL2A1, COL10A1, SOX9, TGF-β3, and TGF-βR3 (scale bar, 200 μm). Data are presented as mean ± SD. *P < 0.05; **P < 0.01; ***P < 0.001. All experiments were performed with 3 independent biological replicates (*n* = 3). MBP-FGF2, maltose-binding protein–fibroblast growth factor 2; MSC, mesenchymal stem cell; qPCR, quantitative polymerase chain reaction; TGF-β1, transforming growth factor-β1; SD, standard deviation.

In T-MSC chondrogenic spheroids, a similar overall pattern was observed. Key chondrogenic markers, SDC2, SOX9, COL2A1, AGN, TGF-β1, and TGF-β3 and their corresponding receptors, were significantly up-regulated in both conditions, regardless of TGF-β1 supplementation. Although exogenous TGF-β1 further amplified their expression, substantial induction was also evident in its absence, indicating robust activation of chondrogenic signaling. Notably, COL1A1 and COL10A1, markers associated with fibrocartilage and hypertrophy, remained low in spheroids cultured without TGF-β1, suggesting that MBP-FGF2-immobilized surfaces effectively promote cartilage-specific lineage commitment while minimizing hypertrophic transition (Fig. 5A). Alcian blue staining revealed GAG accumulation in both groups, although at reduced levels in the absence of TGF-β1 (Fig. 5B). Immunofluorescence staining confirmed the strong expression of COL2A1, colocalized with SOX9, TGF-β3, and TGF-βR3, in both groups. Consistent with observations in H9-MSCs, spheroids differentiated without TGF-β1 showed a lower expression of COL1A1 and COL10A1, indicative of a more cartilage-like phenotype with reduced fibrotic remodeling and hypertrophy. In contrast, TGF-β1-treated spheroids displayed an elevated expression of these markers, consistent with fibrocartilage formation and hypertrophic differentiation (Fig. 5C and D).

Together, these findings demonstrate that MBP-FGF2-mediated surface engineering promotes scaffold-free spheroid formation and localized growth factor signaling, thereby enabling stable chondrogenic maturation in both H9-MSCs and T-MSCs, without exogenous TGF-β1, while suppressing hypertrophic differentiation.

### MBP-FGF2-engineered spheroids undergo in vivo chondrogenic differentiation independent of exogenous TGF-β1

**Fig. 6.**
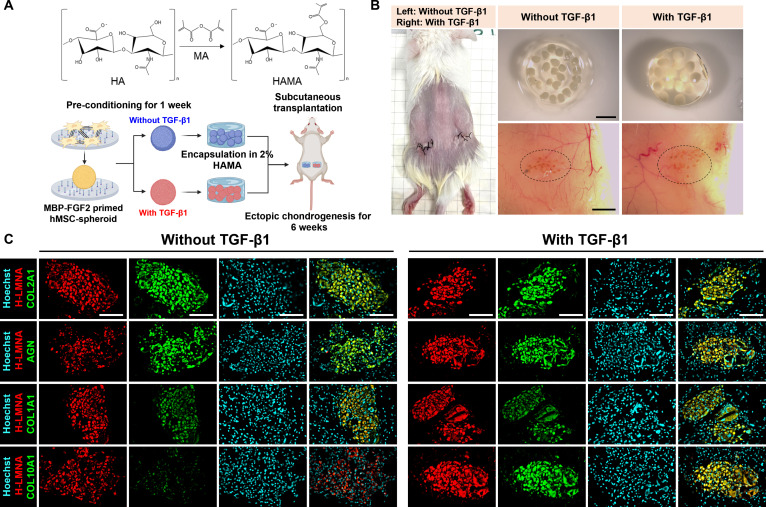
MBP-FGF2 spheroids support in vivo chondrogenic differentiation independent of exogenous TGF-β1. (A) Schematic overview of the experimental design for ectopic chondrogenesis using MBP-FGF2-primed H9-MSC spheroids preconditioned with or without TGF-β1. (B) Photographs of transplanted sites and macroscopic views of explanted grafts at 6 weeks (scale bar, 2 mm). (C) Immunofluorescence staining of explants from spheroids cultured with or without TGF-β1, confirming engraftment of H9-MSCs (H-LMNA, HNA) and robust deposition of cartilage matrix proteins (COL2A1, AGN) with lower expression of COL1A1 and COL10A1 in the absence of TGF-β1 (scale bar, 100 μm). In vivo experiments were conducted with 3 animals per group (*n* = 3). MBP-FGF2, maltose-binding protein–fibroblast growth factor 2; MSC, mesenchymal stem cells; TGF-β1, transforming growth factor-β1.

Our in vitro findings demonstrated that MBP-FGF2-immobilized surfaces promote chondrogenic differentiation even in the absence of exogenous TGF-β1. To evaluate the in vivo chondrogenic potential of pre-differentiated spheroids generated on these surfaces, we performed subcutaneous implantation. Spheroids were preconditioned for 7 d with or without TGF-β1 and encapsulated in 2% (w/v) hyaluronic acid methacrylate (HAMA) hydrogel. The cell-laden constructs were then transplanted into immunodeficient mice, and ectopic chondrogenesis was assessed after 6 weeks (Fig. 6A). At the 6-week endpoint, the HAMA constructs were no longer visually detectable, indicating in vivo biodegradation. However, implantation sites remained discernible, with visible vascular infiltration observed in both groups (Fig. 6B). Mouse body weight remained stable throughout the implantation period, suggesting engraftment and host integration (Fig. S5A). Histological analysis confirmed robust engraftment of human cells, as indicated by immunostaining for H-LMNA. Notably, cartilage-specific proteins COL2A1 and AGN were strongly expressed in both groups (Fig. 6C). These results indicate that spheroids generated on MBP-FGF2-immobilized surfaces retained their chondrogenic phenotype following in vivo transplantation, even in the absence of exogenous TGF-β1. Both COL1A1 and COL10A1 were expressed in explants from both conditions, but their levels were consistently reduced in the absence of exogenous TGF-β1 relative to the TGF-β1-treated groups.

**Fig. 7.**
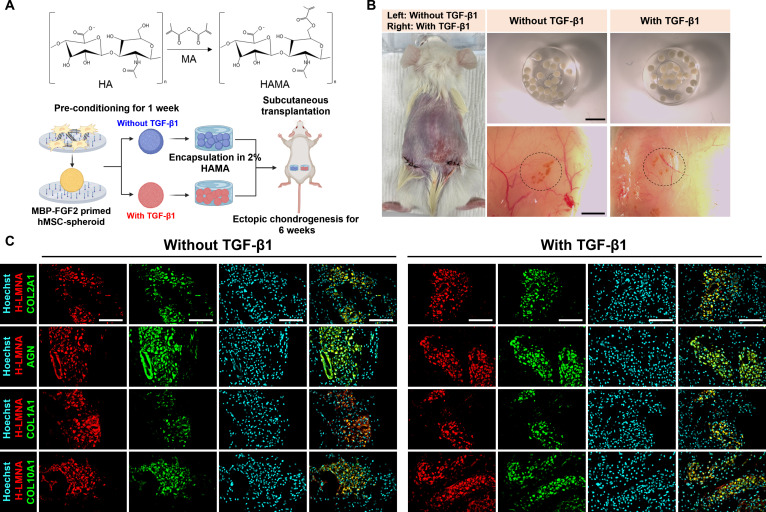
MBP-FGF2 spheroids support in vivo chondrogenic differentiation independent of exogenous TGF-β1. (A) Schematic overview of the experimental design for ectopic chondrogenesis using MBP-FGF2-primed T-MSC spheroids preconditioned with or without TGF-β1. (B) Photographs of transplanted sites and macroscopic views of explanted grafts at 6 weeks (scale bar, 2 mm). (C) Immunofluorescence staining of explants from spheroids cultured with or without TGF-β1, confirming engraftment of T-MSCs (H-LMNA, HNA) and robust deposition of cartilage matrix proteins (COL2A1, AGN) with lower expression of COL1A1 and COL10A1 in the absence of TGF-β1 (scale bar, 100 μm). In vivo experiments were conducted with 3 animals per group (*n* = 3). MBP-FGF2, maltose-binding protein–fibroblast growth factor 2; MSC, mesenchymal stem cells; TGF-β1, transforming growth factor-β1.

To further validate the in vivo potential of T-MSC spheroids generated on MBP-FGF2-immobilized surfaces, we conducted the same transplantation procedure (Fig. 7A). After 6 weeks, the hydrogel constructs were no longer visible. Nevertheless, the implantation sites remained discernible, and gross imaging revealed clear vascular infiltration in both TGF-β1-treated and untreated groups. Comparable levels of vascularization were observed across conditions, and mouse body weight remained stable throughout the implantation period, suggesting successful engraftment and integration with host tissue (Fig. 7B and Fig. S5B). Immunostaining for H-LMNA and HNA immunostaining confirmed the presence of engrafted human cells, alongside strong expression of cartilage-specific proteins COL2A1 and AGN in both groups (Fig. 7C). Both COL1A1 and COL10A1 were expressed in explants from both conditions, but their levels appeared slightly reduced in the absence of exogenous TGF-β1 compared to the TGF-β1-treated groups. Therefore, MBP-FGF2-induced chondrogenic spheroids possess intrinsic functional capacity to form cartilage-like tissue in vivo, even in the absence of sustained exogenous TGF-β1 stimulation. The consistent expression of chondrogenic matrix proteins in both cell types underscores the efficacy of the MBP-FGF2 platform in generating transplantable spheroids capable of autonomous cartilage formation.

## Discussion

TGF-β isoforms, particularly TGF-β1, have long been considered essential for driving MSC chondrogenesis. However, consistent with prior reports, this study shows that prolonged exposure to TGF-β1 promoted hypertrophic and endochondral maturation, limiting its utility for stable hyaline cartilage formation [22]. A central finding of this work is that immobilized MBP-FGF2 provides a biomaterial-based platform that induces spontaneous spheroid formation and drives TGF-β1-independent chondrogenic differentiation of human MSCs (hMSCs). Compared with soluble FGF2, immobilized MBP-FGF2 offers sustained and spatially confined signaling through stable interactions with HSPGs and FGFR1 on the cell surface [23]. This configuration supports integrin-independent adhesion and promotes the formation of uniformly compact spheroids without external forces [24]. By maintaining prolonged FGFR1-mediated signaling, immobilized MBP-FGF2 enhances stem cell function, including improved self-renewal, consistent morphology, and activation of regenerative signaling pathways such as c-Jun N-terminal kinase (JNK) and nuclear factor κB (NF-κB) [21]. These distinct biophysical and biochemical properties position MBP-FGF2 as a bioactive matrix capable of directing stem cell fate beyond the limitations of soluble factor delivery [19]. Another key finding of this study is the early up-regulation of TGF-β3, a well-established regulator of chondrogenesis that synergizes with FGF2 signaling to drive SOX9 activation and COL2A1 expression [25]. The observed increase in the TGF-β3 level in the absence of exogenous TGF-β1 highlights the potential of the MBP-FGF2 platform to harness spontaneous spheroid formation and endogenous signaling mechanisms to promote stable cartilage lineage commitment.

Unlike conventional pellet culture [26], which primarily relies on forced cell aggregation through centrifugation, the MBP-FGF2 interface provides an instructive cell–matrix microenvironment that actively regulates cell behavior. In native cartilage, FGF2 is known to be sequestered within the ECM through binding to HSPGs, forming a matrix-associated reservoir that regulates its local availability [27]. The immobilized MBP-FGF2 surface therefore mimics this physiological mode of growth factor presentation by providing localized and sustained signaling at the cell–matrix interface. Furthermore, interactions between immobilized FGF2 and cell-surface HSPGs can modulate cell–matrix adhesion strength, thereby influencing cytoskeletal organization and cell aggregation behavior [21]. This interaction establishes a biophysical balance between cell–cell and cell–substrate interactions that promote spontaneous and uniform spheroid self-organization without the need for centrifugation-based aggregation [20]. In contrast, conventional pellet culture relies predominantly on centrifugation-induced cell compaction and provides limited control over the biochemical and biophysical cues that regulate cellular organization and function. Importantly, previous studies have shown that spheroid formation on MBP-FGF2 surfaces enhances the secretion of pro-angiogenic and regenerative factors, including vascular endothelial growth factor (VEGF), hepatocyte growth factor (HGF), and interleukin-8 (IL-8), indicating improved functional activity of stem cells in 3D culture [21]. Such enhanced paracrine signaling has been associated with improved therapeutic outcomes in ischemic disease models.

Taken together, the MBP-FGF2 interface functions not only as a platform for cell aggregation but also as a bioinstructive matrix that regulates cell–matrix interactions, promotes controlled 3D self-organization, and enhances stem cell functionality. These features distinguish the MBP-FGF2 platform from conventional pellet-based aggregation methods and highlight its potential as a physiologically relevant strategy for generating functional stem cell spheroids.

Our findings demonstrate that MBP-FGF2-engineered surfaces provide integrated biophysical and biochemical cues that activate early TGF-β signaling and initiates chondrogenic lineage priming without the need for exogeneous TGF-β1. The chondrogenic phenotype was preserved in vivo, underscoring the therapeutic potential of this platform for cartilage tissue engineering. To enhance the translational relevance of this study, we employed both H9-MSCs and T-MSCs. H9-MSCs provide a homogeneous and highly expandable and standardized cell source. Previous studies have shown that hESC-derived MSCs exhibit robust proliferative capacity compared with adult tissue-derived MSCs [28]. In addition, H9-MSCs have been reported to possess immunoprivileged and immunomodulatory properties comparable to bone marrow-derived MSCs while showing lower human leukocyte antigen (HLA)-DR expression and reduced responsiveness to inflammatory induction, supporting their potential utility in allogeneic applications [29]. T-MSCs possess several advantages compared with MSCs obtained from conventional sources such as bone marrow or adipose tissue. These cells can be isolated from tonsillar tissues collected during tonsillectomy procedures, which are discarded after surgery, thereby providing an easily accessible cell source with strong regenerative capacity and clinical applicability [30]. Previous studies have shown that T-MSCs display relatively rapid proliferation during early culture and maintain this proliferative capacity across extended passages compared with bone marrow-derived MSCs and adipose-derived MSCs [31,32]. In addition, the proliferative and differentiation capacities of T-MSCs appear to be relatively stable across donors of different ages. Importantly, these functional properties are largely preserved after cryopreservation, supporting the feasibility of long-term storage and biobanking for potential therapeutic applications. From a chondrogenic standpoint, both cell types have previously demonstrated the ability to differentiate into cartilage-like cells under defined inductive conditions [33]. These complementary properties highlight the robustness and versatility of the MBP-FGF2 platform across distinct stem cell sources for both in vitro and in vivo applications.

The consistent responses observed across both cell types, including spheroid formation, early activation of TGF-β signaling, sustained expression of cartilage-specific markers, and reduced hypertrophic transition, highlight the robustness and generalizability of the MBP-FGF2 platform for cartilage tissue engineering. To further elucidate the molecular mechanisms underlying early spheroid formation on MBP-FGF2-immobilized surfaces, we examined early transcriptional changes following cell adhesion. RNA-seq analysis performed at 4 h revealed up-regulation of FGF receptor-associated genes and TGF-β3, along with enrichment of gene sets related to chondrocyte development and ECM organization. These findings were further validated using qPCR, which confirmed time-dependent increases in SOX9, COL2A1, AGN, and TGF-β3 expression in spheroids derived from both cell types. These observations suggest that immobilized MBP-FGF2 is associated with early chondrogenic priming, potentially through engagement of FGF–HSPG–FGFR signaling and modulation of endogenous TGF-β3–related pathways. The observed up-regulation of SOX9 and TGF-β3, which are widely recognized regulators of cartilage development, supports this interpretation. Rather than acting as a lineage-deterministic factor, MBP-FGF2 appears to provide a microenvironment that is permissive for chondrogenic commitment under the specific culture conditions used in this study. These results align with previous studies demonstrating that TGF-β3 is a potent inducer of hyaline-like cartilage from MSCs, and that its early activation can be mediated through FGF-HSPG-dependent pathways, even in the absence of exogenous TGF-β1 [8]. Emerging evidence supports a synergistic relationship between FGF2 and TGF-β3 during chondrogenesis. In bone marrow-derived mesenchymal stem cells (BM-MSCs), costimulation with FGF2 and TGF-β3 significantly enhanced both early and late chondrogenic marker expression [25]. Similarly, in synovial chondromatosis models, FGF2 treatment elevated endogenous TGF-β3 levels, suggesting that FGF2 may act upstream to modulate TGF-β3-mediated signaling [34]. Moreover, FGF2 priming has been shown to increase basal SOX9 expression in MSCs, thereby enhancing chondrogenic differentiation even in the absence of TGF-β1 [35]. Taken together, these findings suggest that FGF2 functions as an upstream modulator of early chondrogenic priming, capable of contributing to endogenous TGF-β3 signaling and enhancing basal SOX9 expression. Among the 23 members of the FGF family, FGF2 was selected based on its well-established role in mesenchymal stem cell expansion, maintenance of multipotency, and early chondrogenic priming. In addition, FGF2 exhibits strong affinity for HSPGs, enabling stable immobilization and sustained receptor engagement through the HSPG–FGFR axis [36]. These characteristics support the interpretation that immobilized FGF2 can provide localized and sustained signaling, which may facilitate early chondrogenic responses rather than directly instruct lineage specification.

Supporting this mechanism, we observed up-regulation of SDC2, a key member of the HSPG family that functions as a co-receptor facilitating FGF2–FGFR1 interactions and stabilizing ligand–receptor complex formation [37]. HSPGs play a critical role in chondrogenesis by regulating the availability and activity of heparin-binding growth factors, including FGF and bone morphogenetic protein (BMP), thereby enhancing chondrocyte differentiation and ECM production [38,39]. The concurrent up-regulation of SDC2 in our RNA-seq and qPCR analyses reinforces a model in which immobilized FGF2 activates HSPG-mediated signaling. Such activation may contribute to the induction of endogenous TGF-β3 expression and subsequent chondrogenic commitment. This mechanism is consistent with previous reports indicating that FGF2–HSPG interactions can induce endogenous TGF-β3 expression and support chondrogenesis [38].

Since hypertrophic cartilage and fibrocartilage are prone to degeneration and ossification, the formation of hyaline cartilage is essential for effective functional restoration [40]. Fibrocartilage, characterized by elevated COL1A1 expression, often develops in response to trauma or degenerative injury. However, compared with native hyaline cartilage, fibrocartilage tissue exhibits inferior mechanical properties and is more susceptible to tearing or breakdown under load, ultimately compromising joint function [41]. In parallel, hypertrophic differentiation, marked by COL10A1 expression, represents a transitional stage toward endochondral ossification, in which hypertrophic chondrocytes undergo apoptosis and are replaced by bone tissue [2]. Although this process is essential for skeletal development, it is undesirable in articular cartilage repair due to its tendency to accelerate ossification and destabilize the graft, as evidenced by studies showing that induced pluripotent stem cell (iPSC)-derived chondrocytes with low COL10A1 expression have demonstrated stable cartilage repair in vivo, whereas bone marrow-derived mesenchymal stem cell (BMSC)-derived chondrocytes with higher COL10A1 levels exhibit hypertrophic features associated with inferior outcomes [42]. Similarly, conventional MSC pellet cultures often display elevated COL1A1 and COL10A1 expression, indicative of fibrocartilage or hypertrophic maturation, even under defined chondrogenic conditions [43]. The ability to induce robust COL2A1 and AGN expression while minimizing hypertrophic marker expression represents a key improvement over conventional MSC-based chondrogenesis protocols, which often rely on prolonged TGF-β1 exposure. Such protocols often lead to terminal hypertrophic differentiation, limiting their clinical applicability [22]. Previous studies have demonstrated that controlling hypertrophy is a critical requirement for achieving stable and functional cartilage regeneration. For example, nanofibrous poly(l-lactic acid) scaffolds combined with Matrilin-3 have been shown to attenuate mesenchymal stem cell (MSC) hypertrophy and inhibit endochondral ossification, thereby improving the stability of regenerated cartilage tissue [44]. Similarly, 3D polycaprolactone scaffolds conjugated with BMP-2 were reported to support cartilage formation without promoting excessive hypertrophic maturation in both in vitro and in vivo settings [45]. Collectively, these findings emphasize that, in MSC-based cartilage engineering, attenuation, rather than complete elimination, of hypertrophic progression is a realistic and important objective for maintaining a stable cartilage phenotype.

In this context, our MBP-FGF2 platform appears to support chondrogenic matrix formation while limiting hypertrophic progression. Specifically, localized FGF2 presentation via MBP may facilitate receptor-mediated signaling through FGF2–HSPG–FGFR interactions, which has been associated with the regulation of early chondrogenic events and modulation of TGF-β signaling. Consistent with our in vivo findings, cartilage tissues formed under these conditions exhibited robust expression of cartilage matrix components alongside relatively low levels of hypertrophic markers, supporting a “hyaline-like” cartilage phenotype rather than fully mature hyaline cartilage. Notably, although both H9-MSCs and T-MSCs responded effectively to MBP-FGF2-mediated chondrogenic cues, subtle differences in hypertrophic marker expression were observed between the 2 cell types. These differences may reflect intrinsic variations in developmental origin and epigenetic priming. Previous studies have shown that iPSC-derived mesenchymal progenitors possess distinct transcriptomic profiles compared with somatic tissue-derived MSCs, which can influence chondrogenic maturation and susceptibility to hypertrophic progression [46]. In addition, donor-dependent variability has been reported to affect MSC differentiation behavior even within the same tissue source [47]. Taken together, these factors may contribute to the differences in hypertrophic tendencies observed in our study.

**Fig. 8.**
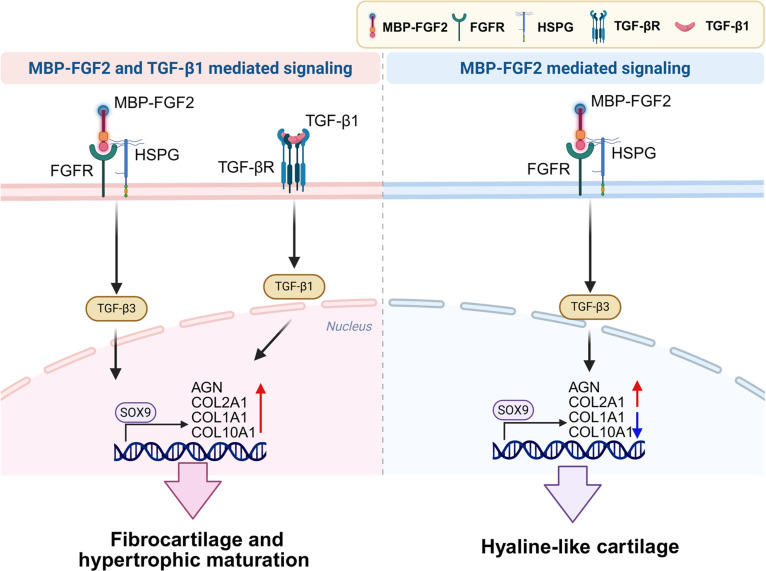
Schematic illustration of chondrogenic differentiation pathways under dual MBP-FGF2 and TGF-β1 stimulation (left) versus MBP-FGF2 stimulation alone (right). Dual signaling promotes fibrocartilage formation and hypertrophic maturation with increased COL1A1 and COL10A1 expression, whereas MBP-FGF2 alone induces a hyaline-like phenotype with strong COL2A1 and AGN expression and reduced hypertrophic marker expression. MBP-FGF2, maltose-binding protein–fibroblast growth factor 2; MSC, mesenchymal stem cells; TGF-β1, transforming growth factor-β1.

This mechanism promotes strong expression of COL2A1 and AGN, while suppressing fibrocartilage- and hypertrophy-associated markers, enabling the formation of a stable hyaline-like cartilage phenotype without the need for exogenous TGF-β1 (Fig. 8). Mechanistically, the TGF-β1-independent chondrogenic differentiation observed in MBP-FGF2 spheroids likely results from a combination of reduced cell–substrate adhesion and enhanced cell–cell interactions, which together mimic mesenchymal condensation, a characteristic of early cartilage development. This niche-like microenvironment activates chondrogenic transcription factors and synergizes with FGF2–HSPG–FGFR signaling pathways, thus triggering endogenous TGF-β pathways. These findings align with emerging evidence suggesting that well-defined, biomaterial-driven microenvironments engage endogenous signaling mechanisms to support in vivo cartilage formation in the absence of exogenous TGF-β1 [48]. Consistent with this, our in vivo results demonstrated that spheroids preconditioned on MBP-FGF2-coated surfaces maintained a cartilage-like phenotype, characterized by robust COL2A1 and AGN deposition. COL10A1 levels remained low in the absence of exogenous TGF-β1, indicating that the MBP-FGF2 platform supports cartilage-specific matrix formation and effectively suppresses hypertrophic differentiation. Although COL10A1 expression was detectable, its levels remained relatively low in the absence of exogenous TGF-β1, particularly compared with the TGF-β1-treated group. This pattern suggests limited hypertrophic progression rather than complete suppression. In line with previous reports, stabilization of MSC-derived cartilage is generally achieved through attenuation, rather than complete elimination, of hypertrophy [49]. For example, biomaterial-based strategies and modulation of TGF-β signaling have been shown to reduce COL10A1 expression while preserving cartilage matrix deposition, thereby improving phenotypic stability following in vivo implantation [50]. Taken together, these findings highlight the potential of the MBP-FGF2 platform to support stable hyaline-like cartilage formation in vivo without exogenous TGF-β1 while limiting hypertrophic progression relative to TGF-β1-treated group.

Our results suggest that MBP-FGF2 surface engineering provides both mechanical and biochemical signals necessary to drive MSCs toward a chondrogenic lineage. The activation of FGF2–HSPG-mediated cell adhesion was accompanied by reduced cell–substrate adhesion and enhanced spheroid formation. Notably, chondrogenic differentiation occurred in the absence of exogenous TGF-β1, suggesting that early, localized signaling via MBP-FGF2 is sufficient to initiate lineage commitment. This approach enabled the production of cartilage-specific matrix components and minimized fibrocartilage formation and hypertrophic differentiation.

Building on these findings, this study presents a biomaterial-based strategy that leverages spatially confined, receptor-specific FGF2 signaling to induce spontaneous spheroid formation and chondrogenic differentiation of hMSCs without exogenous TGF-β1. MBP-FGF2-immobilized surfaces mimic key features of developmental mesenchymal condensation by reducing cell–matrix adhesion, enhancing cell–cell interactions, and activating endogenous TGF-β3 signaling through an HSPG–FGF2-dependent mechanism. This coordinated biophysical and biochemical modulation drives robust expression of cartilage-specific matrix proteins (COL2A1 and AGN) while suppressing hypertrophic and fibrocartilage-associated markers (COL10A1 and COL1A1) in the absence of sustained TGF-β1 exposure. Collectively, these findings establish MBP-FGF2 surface engineering as a robust, bioinstructive interface that drives stable hyaline-like cartilage formation without exogenous TGF-β1, supporting a defined, growth factor-free approach for advancing the field of cartilage tissue engineering. While ectopic cartilage-like tissues and stable engraftment were observed following subcutaneous implantation, this model does not fully recapitulate the native articular cartilage environment. Therefore, further evaluation in clinically relevant orthotopic cartilage defect models will be necessary to assess functional integration, mechanical performance, and long-term stability under physiologically relevant conditions. Such studies will be important to determine the translational potential of this platform for cartilage repair.

## Data Availability

All data generated or analyzed during the present study could be found within the manuscript and the supplemental files.

## References

[1] Guo X, Xi L, Yu M, Fan Z, Wang W, Ju A, Liang Z, Zhou G, Ren W. Regeneration of articular cartilage defects: Therapeutic strategies and perspectives. J Tissue Eng. 2023;14:20417314231164765. 37025158 10.1177/20417314231164765 PMC10071204

[2] Armiento AR, Alini M, Stoddart MJ. Articular fibrocartilage—Why does hyaline cartilage fail to repair? Adv Drug Deliv Rev. 2019;146:289–305. 30605736 10.1016/j.addr.2018.12.015

[3] Chu H, Zhang S, Zhang Z, Yue H, Liu H, Li B, Yin F. Comparison studies identify mesenchymal stromal cells with potent regenerative activity in osteoarthritis treatment. npj Regener Med. 2024;9(1):14. 10.1038/s41536-024-00358-y PMC10984924 38561335

[4] Johnstone B, Hering TM, Caplan AI, Goldberg VM, Yoo JU. In vitro chondrogenesis of bone marrow-derived mesenchymal progenitor cells. Exp Cell Res. 1998;238(1):265–272. 9457080 10.1006/excr.1997.3858

[5] Mackay AM, Beck SC, Murphy JM, Barry FP, Chichester CO, Pittenger MF. Chondrogenic differentiation of cultured human mesenchymal stem cells from marrow. Tissue Eng. 1998;4(4):415–428. 9916173 10.1089/ten.1998.4.415

[6] Zhang L, Su P, Xu C, Yang J, Yu W, Huang D. Chondrogenic differentiation of human mesenchymal stem cells: A comparison between micromass and pellet culture systems. Biotechnol Lett. 2010;32(9):1339–1346. 20464452 10.1007/s10529-010-0293-x

[7] Varghese S, Hwang NS, Canver AC, Theprungsirikul P, Lin DW, Elisseeff J. Chondroitin sulfate based niches for chondrogenic differentiation of mesenchymal stem cells. Matrix Biol. 2008;27(1):12–21. 17689060 10.1016/j.matbio.2007.07.002

[8] Du X, Cai L, Xie J, Zhou X. The role of TGF-beta3 in cartilage development and osteoarthritis. Bone Res. 2023;11(1):2. 36588106 10.1038/s41413-022-00239-4 PMC9806111

[9] Pelttari K, Steck E, Richter W. The use of mesenchymal stem cells for chondrogenesis. Injury. 2008;39(1, Supplement):58–65. 10.1016/j.injury.2008.01.038 18313473

[10] Pelttari K, Winter A, Steck E, Goetzke K, Hennig T, Ochs BG, Aigner T, Richter W. Premature induction of hypertrophy during in vitro chondrogenesis of human mesenchymal stem cells correlates with calcification and vascular invasion after ectopic transplantation in SCID mice. Arthritis Rheum. 2006;54(10):3254–3266. 17009260 10.1002/art.22136

[11] Chiou M, Xu Y, Longaker MT. Mitogenic and chondrogenic effects of fibroblast growth factor-2 in adipose-derived mesenchymal cells. Biochem Biophys Res Commun. 2006;343(2):644–652. 16554022 10.1016/j.bbrc.2006.02.171

[12] Handorf AM, Li WJ. Fibroblast growth factor-2 primes human mesenchymal stem cells for enhanced chondrogenesis. PLOS ONE. 2011;6(7): Article e22887. 21818404 10.1371/journal.pone.0022887 PMC3144950

[13] Ornitz DM, Marie PJ. FGF signaling pathways in endochondral and intramembranous bone development and human genetic disease. Genes Dev. 2002;16(12):1446–1465. 12080084 10.1101/gad.990702

[14] Stewart AA, Byron CR, Pondenis H, Stewart MC. Effect of fibroblast growth factor-2 on equine mesenchymal stem cell monolayer expansion and chondrogenesis. Am J Vet Res. 2007;68(9):941–945. 17764407 10.2460/ajvr.68.9.941

[15] Adesida AB, Grady LM, Khan WS, Hardingham TE. The matrix-forming phenotype of cultured human meniscus cells is enhanced after culture with fibroblast growth factor 2 and is further stimulated by hypoxia. Arthritis Res Ther. 2006;8(3):R61. 16563175 10.1186/ar1929 PMC1526627

[16] Mishra A, Modi U, Sharma R, Bhatia D, Solanki R. Biochemical and biophysical cues of the extracellular matrix modulates stem cell fate: Progress and prospect in extracellular matrix mimicking biomaterials. Biomed Eng Adv. 2025;9: Article 100143.

[17] Sung TC, Pan ZX, Wang T, Lin HY, Chang CL, Hung LC, Subbiah SK, Renuka RR, Chou SJ, Chiou SH, et al. Material surface conjugated with fibroblast growth factor-2 for pluripotent stem cell culture and differentiation. Regener Biomater. 2025;12:rbaf003. 10.1093/rb/rbaf003 PMC11835233 39967781

[18] Shakya A, Imado E, Nguyen PK, Matsuyama T, Horimoto K, Hirata I, Kato K. Oriented immobilization of basic fibroblast growth factor: Bioengineered surface design for the expansion of human mesenchymal stromal cells. Sci Rep. 2020;10(1):8762. 32472000 10.1038/s41598-020-65572-2 PMC7260242

[19] Kang JM, Han M, Park IS, Jung Y, Kim SH, Kim S-H. Adhesion and differentiation of adipose-derived stem cells on a substrate with immobilized fibroblast growth factor. Acta Biomater. 2012;8(5):1759–1767. 22285427 10.1016/j.actbio.2012.01.005

[20] Jeong JH, Park KN, Kim JH, Noh K, Hur SS, Kim Y, Hong M, Chung JC, Park JH, Lee J, et al. Self-organized insulin-producing beta-cells differentiated from human omentum-derived stem cells and their in vivo therapeutic potential. Biomater Res. 2023;27(1):82. 37644502 10.1186/s40824-023-00419-1 PMC10466773

[21] Choi J, Choi W, Joo Y, Chung H, Kim D, Oh SJ, Kim SH. FGF2-primed 3D spheroids producing IL-8 promote therapeutic angiogenesis in murine hindlimb ischemia. npj Regener Med. 2021;6(1):48. 10.1038/s41536-021-00159-7 PMC8373896 34408157

[22] Futrega K, Robey PG, Klein TJ, Crawford RW, Doran MR. A single day of TGF-beta1 exposure activates chondrogenic and hypertrophic differentiation pathways in bone marrow-derived stromal cells. Commun Biol. 2021;4(1):29. 33398032 10.1038/s42003-020-01520-0 PMC7782775

[23] Kang J, Park HM, Kim YW, Kim YH, Varghese S, Seok HK, Kim YG, Kim SH. Control of mesenchymal stem cell phenotype and differentiation depending on cell adhesion mechanism. Eur Cell Mater. 2014;28:387–403. 25422949 10.22203/ecm.v028a27

[24] Hong S, Oh SJ, Choi D, Hwang Y, Kim SH. Self-organized liver microtissue on a bio-functional surface: The role of human adipose-derived stromal cells in hepatic function. Int J Mol Sci. 2020;21(13). 10.3390/ijms21134605 PMC7369942 32610471

[25] Stamnitz S, Krawczenko A, Klimczak A. Combined TGF-beta3 and FGF-2 stimulation enhances chondrogenic potential of ovine bone marrow-derived MSCs. Cells. 2025;14(13): Article 1013. 40643533 10.3390/cells14131013 PMC12249412

[26] Hsieh CC, Chang CC, Hsu PJ, Chen L, Yen BL. Protocol for efficient human MSC chondrogenesis via Wnt antagonism instead of TGF-beta. STAR Protoc. 2023;4(4): Article 102728. 37979177 10.1016/j.xpro.2023.102728 PMC10694580

[27] Vincent TL, McLean CJ, Full LE, Peston D, Saklatvala J. FGF-2 is bound to perlecan in the pericellular matrix of articular cartilage, where it acts as a chondrocyte mechanotransducer. Osteoarthr Cartil. 2007;15(7):752–763. 10.1016/j.joca.2007.01.021 17368052

[28] Brown PT, Squire MW, Li WJ. Characterization and evaluation of mesenchymal stem cells derived from human embryonic stem cells and bone marrow. Cell Tissue Res. 2014;358(1):149–164. 24927918 10.1007/s00441-014-1926-5 PMC4329984

[29] Fu X, Chen Y, Xie FN, Dong P, Liu WB, Cao Y, Zhang WJ, Xiao R. Comparison of immunological characteristics of mesenchymal stem cells derived from human embryonic stem cells and bone marrow. Tissue Eng Part A. 2015;21(3-4):616–626. 25256849 10.1089/ten.tea.2013.0651 PMC4334098

[30] Kim HY, Yang J, Kim HS, Jung SY. Stepwise differentiation of airway epithelial cells from human tonsil-derived mesenchymal stem cells. Stem Cell Res Ther. 2025;16(1):354. 40624536 10.1186/s13287-025-04397-0 PMC12235983

[31] Janjanin S, Djouad F, Shanti RM, Baksh D, Gollapudi K, Prgomet D, Rackwitz L, Joshi AS, Tuan RS. Human palatine tonsil: A new potential tissue source of multipotent mesenchymal progenitor cells. Arthritis Res Ther. 2008;10(4):R83. 18662393 10.1186/ar2459 PMC2575631

[32] Kim SY, Kim YR, Park WJ, Kim HS, Jung SC, Woo SY, Jo I, Ryu KH, Park JW. Characterisation of insulin-producing cells differentiated from tonsil derived mesenchymal stem cells. Differentiation. 2015;90(1-3):27–39. 26391447 10.1016/j.diff.2015.08.001

[33] Park J, Kim IY, Patel M, Moon HJ, Hwang S-J, Jeong B. 2D and 3D hybrid systems for enhancement of chondrogenic differentiation of tonsil-derived mesenchymal stem cells. Adv Funct Mater. 2015;25(17):2573–2582.

[34] Li Y, El Mozen LA, Cai H, Fang W, Meng Q, Li J, Deng M, Long X. Transforming growth factor beta 3 involved in the pathogenesis of synovial chondromatosis of temporomandibular joint. Sci Rep. 2015;5:8843. 25742744 10.1038/srep08843 PMC4351526

[35] Cucchiarini M, Madry H, Ma C, Thurn T, Zurakowski D, Menger MD, Kohn D, Trippel SB, Terwilliger EF. Improved tissue repair in articular cartilage defects in vivo by rAAV-mediated overexpression of human fibroblast growth factor 2. Mol Ther. 2005;12(2):229–238. 16043094 10.1016/j.ymthe.2005.03.012

[36] Xue S, Zhou F, Zhao T, Zhao H, Wang X, Chen L, Li JP, Luo SZ. Phase separation on cell surface facilitates bFGF signal transduction with heparan sulphate. Nat Commun. 2022;13(1):1112. 35236856 10.1038/s41467-022-28765-z PMC8891335

[37] Park PW, Reizes O, Bernfield M. Cell surface heparan sulfate proteoglycans: Selective regulators of ligand-receptor encounters. J Biol Chem. 2000;275(39):29923–29926. 10931855 10.1074/jbc.R000008200

[38] Chen J, Wang Y, Chen C, Lian C, Zhou T, Gao B, Wu Z, Xu C. Exogenous heparan sulfate enhances the TGF-beta3-induced chondrogenesis in human mesenchymal stem cells by activating TGF-beta/Smad signaling. Stem Cells Int. 2016;2016:1520136. 26783399 10.1155/2016/1520136 PMC4691498

[39] Otsuki S, Hanson SR, Miyaki S, Grogan SP, Kinoshita M, Asahara H, Wong CH, Lotz MK. Extracellular sulfatases support cartilage homeostasis by regulating BMP and FGF signaling pathways. Proc Natl Acad Sci USA. 2010;107(22):10202–10207. 20479257 10.1073/pnas.0913897107 PMC2890424

[40] Li J, Jiang H, Tan G, Lv Z, Liu Z, Guo H, Sun Z, Xu X, Shi D. Fibrocartilage hyalinization: A potential therapeutic strategy for articular fibrocartilage. J Orthop Translat. 2025;52:313–324. 40421144 10.1016/j.jot.2025.04.013 PMC12104164

[41] Krakowski P, Rejniak A, Sobczyk J, Karpinski R. Cartilage integrity: A review of mechanical and frictional properties and repair approaches in osteoarthritis. Healthcare. 2024;12(16): Article 1648. 39201206 10.3390/healthcare12161648 PMC11353818

[42] Lee MS, Lin EC, Sivapatham A, Leiferman EM, Jiao H, Lu Y, Nemke BW, Leiferman M, Markel MD, Li WJ. Autologous iPSC- and MSC-derived chondrocyte implants for cartilage repair in a miniature pig model. Stem Cell Res Ther. 2025;16(1):86. 39988676 10.1186/s13287-025-04215-7 PMC11849328

[43] Mueller MB, Tuan RS. Functional characterization of hypertrophy in chondrogenesis of human mesenchymal stem cells. Arthritis Rheum. 2008;58(5):1377–1388. 18438858 10.1002/art.23370 PMC3612425

[44] Liu Q, Wang J, Chen Y, Zhang Z, Saunders L, Schipani E, Chen Q, Ma PX. Suppressing mesenchymal stem cell hypertrophy and endochondral ossification in 3D cartilage regeneration with nanofibrous poly(l-lactic acid) scaffold and matrilin-3. Acta Biomater. 2018;76:29–38. 29940371 10.1016/j.actbio.2018.06.027 PMC6086372

[45] Jeong CG, Zhang H, Hollister SJ. Three-dimensional polycaprolactone scaffold-conjugated bone morphogenetic protein-2 promotes cartilage regeneration from primary chondrocytes in vitro and in vivo without accelerated endochondral ossification. J Biomed Mater Res A. 2012;100(8):2088–2096. 22615065 10.1002/jbm.a.33249

[46] Khan NM, Doan TN, Kaiser JM, Drissi H. Chondrogenic potential of mesenchymal progenitors from somatic and cartilage-derived iPSCs is predicted by their transcriptomic signatures. Genes Dis. 2026;13(2): Article 101730. 41492477 10.1016/j.gendis.2025.101730 PMC12765249

[47] Kim M, Erickson IE, Huang AH, Garrity ST, Mauck RL, Steinberg DR. Donor variation and optimization of human mesenchymal stem cell chondrogenesis in hyaluronic acid. Tissue Eng Part A. 2018;24(21-22):1693–1703. 29792383 10.1089/ten.tea.2017.0520 PMC6238652

[48] Udomluck N, Park H, Lee JY. Advances in biomaterial-based composite spheroid for articular cartilage regeneration. J Tissue Eng. 2025;16:20417314251349669. 40626181 10.1177/20417314251349669 PMC12231992

[49] Deng Y, Lei G, Lin Z, Yang Y, Lin H, Tuan RS. Engineering hyaline cartilage from mesenchymal stem cells with low hypertrophy potential via modulation of culture conditions and Wnt/beta-catenin pathway. Biomaterials. 2019;192:569–578. 30544046 10.1016/j.biomaterials.2018.11.036 PMC6733256

[50] Zhao C, Li X, Han X, Li Z, Bian S, Zeng W, Ding M, Liang J, Jiang Q, Zhou Z, et al. Molecular co-assembled strategy tuning protein conformation for cartilage regeneration. Nat Commun. 2024;15(1):1488. 38374253 10.1038/s41467-024-45703-3 PMC10876949

