# Supplementary material for: Fibroblast Growth Factor 2-Immobilized Biointerfaces Drive Exogenous Transforming Growth Factor β1-Independent Chondrogenesis of Human Mesenchymal Stem Cell and Ectopic Cartilage Tissue Formation
Source: Biomater Res. 2026 Jun 1;30:0367. doi: 10.34133/bmr.0367 (PMC13223357; doi:10.34133/bmr.0367)
Supplement: Supplementary 1 — Figs. S1 to S5 Table S1 [file bmr.0367.f1.zip › Table_FINAL.docx]

Supplementary Materials

Fibroblast Growth Factor 2-immobilized biointerfaces drive exogenous transforming growth factor beta 1-independent chondrogenesis of human mesenchymal stem cell and ectopic cartilage tissue formation

Ji Hoon Jeong^1,2,†^, Jae Hong Park^3,†^, Myung Jin Ban^3^, Yeongrok Lee^3^, Hyeongtae Kim^3^, Yura Kim^3^, Gahyun Kim^1,2^, Sai-Won Kwon^4^, Ju Hun Lee^5^, Sang-Heon Kim^6,7,*^, and Yongsung Hwang^1,2,*^

*^1^Soonchunhyang Institute of Medi-Bio Science (SIMS), Soonchunhyang University, Cheonan 31151, Chungnam-do, Republic of Korea
^2^Department of Integrated Biomedical Science, Soonchunhyang University, Asan 31538, Chungnam-do, Republic of Korea*

*^3^Department of Otorhinolaryngology–Head and Neck Surgery, College of Medicine, Soonchunhyang University Cheonan Hospital, Cheonan 31151, Republic of Korea*

*^4^Department of Orthopaedic Surgery, College of Medicine, Soonchunhyang University Cheonan Hospital, Cheonan 31151, Republic of Korea*

*^5^Department of Bionano Engineering, Center for Bionano Intelligence Education and Research, Hanyang University, Ansan 15588, Republic of Korea*

*^6^Center for Biomaterials, Biomedical Research Institute, Korea Institute of Science and Technology, Seoul 02792, Republic of Korea*

*^7^Department of Bio-Med Engineering, KIST School, Korea University of Science and Technology, Seoul 02792, Republic of Korea*

**Address correspondence to:* [skimbrc@kist.re.kr](mailto:skimbrc@kist.re.kr) (S.K.); [yshwang0428@sch.ac.kr](mailto:yshwang0428@sch.ac.kr) (Y.H.)

*^†^These authors contributed equally to this work.*

**Table 1.** List of primers used for quantitative PCR

| Gene | Primer Sequence (5′–3′) |
| --- | --- |
| *GAPDH* | F-CAC TCC ACC TTT GAC GC |
|  | R-GGT CCA GGG GTC TTA CTC C |
| *SOX9* | F-AGC GAA CGC ACA TCA AGA C |
|  | R-CTG TAG GCG ATC TGT TGG GG |
| *COL2A1* | F-GGC AAT AGC AGG TTC ACG TAC A |
|  | R-CGA TAA CAG TCT TGC CCC ACT T |
| *COL1A1* | F-CAA GAC AGT GAT TGA ATA CAA AAC CA |
|  | R-ACG TCG AAG CCG AAT TCC T |
| *COL10A1* | F-CAA GGC ACC ATC TCC AGG AA |
|  | R-AAA GGG TAT TTG TGG CAG CAT ACT |
| *AGN* | F-CCC CTG CTA TTT CAT CGA CCC |
|  | R-GAC ACA CGG CTC CAC TTG AT |
| *TGF-β1* | F-CTA ATG GTG GAA ACC CAC AAC G |
|  | R-TAT CGC CAG GAA TTG TTG CTG |
| *TNXB* | F-GCC CTG CTC ACT TGG ACT G |
|  | R-GGA GCC GTG CAT TGT AGG AG |
| *SDC2* | F-GTG GAT CCT GCT CAC CTT GG |
|  | R-CAG ACG CAG AAG CGT AGT CA |
| *TGF-β3* | F-ACT TGC ACC ACC TTG GAC TTC |
|  | R-GGT CAT CAC CGT TGG CTC A |
| *TGF-β3R* | F-TGG GGT CTC CAG ACT GTT TTT |
|  | R-CTG CTC CAT ACT CTT TTC GGG |

Abbreviations: GAPDH, glyceraldehyde-3-phosphate dehydrogenase; SOX9, SRY-box transcription factor 9; COL2A1, collagen type II alpha 1; COL1A1, collagen type I alpha 1; COL10A1, collagen type X alpha 1; AGN, aggrecan; TGF-β1, transforming growth factor beta 1; TNXB, tenascin; SDC2, syndecan-2; TGF-β3, transforming growth factor beta 3; TGF-β3R, transforming growth factor beta receptor 3.
